# Meclozine Attenuates the MARK Pathway in Mammalian Chondrocytes and Ameliorates FGF2-Induced Bone Hyperossification in Larval Zebrafish

**DOI:** 10.3389/fcell.2021.694018

**Published:** 2022-01-18

**Authors:** Genta Takemoto, Masaki Matsushita, Takaaki Okamoto, Toshinari Ito, Yuki Matsuura, Chieko Takashima, Toyofumi Fengshi Chen-Yoshikawa, Hiromichi Ebi, Shiro Imagama, Hiroshi Kitoh, Kinji Ohno, Yasuyuki Hosono

**Affiliations:** ^1^ Department of Orthopaedic Surgery, Nagoya University Graduate School of Medicine, Nagoya, Japan; ^2^ Division of Neurogenetics, Center for Neurological Diseases and Cancer, Nagoya University Graduate School of Medicine, Nagoya, Japan; ^3^ Division of Molecular Therapeutics, Aichi Cancer Center Research Institute, Nagoya, Japan; ^4^ Department of Thoracic Surgery, Nagoya University Graduate School of Medicine, Nagoya, Japan; ^5^ Department of Orthopaedic Surgery, Aichi Children’s Health and Medical Center, Obu, Japan; ^6^ Department of Comprehensive Pediatric Medicine, Nagoya University Graduate School of Medicine, Nagoya, Japan; ^7^ Department of Pharmacology, Okayama University Graduate School of Medicine, Dentistry and Pharmaceutical Sciences, Okayama, Japan

**Keywords:** FGFR3, achondroplasia, meclozine, zebrafish, bone

## Abstract

Meclozine has been developed as an inhibitor of fibroblast growth factor receptor 3 (FGFR3) to treat achondroplasia (ACH). Extracellular signal regulated kinase (ERK) phosphorylation was attenuated by meclozine in FGF2-treated chondrocyte cell line, but the site of its action has not been elucidated. Although orally administered meclozine promoted longitudinal bone growth in a mouse model of ACH, its effect on craniofacial bone development during the early stage remains unknown. Herein, RNA-sequencing analysis was performed using murine chondrocytes from FGF2-treated cultured tibiae, which was significantly elongated by meclozine treatment. Gene set enrichment analysis demonstrated that FGF2 significantly increased the enrichment score of mitogen-activated protein kinase (MAPK) family signaling cascades in chondrocytes; however, meclozine reduced this enrichment. Next, we administered meclozine to FGF2-treated larval zebrafish from 8 h post-fertilization (hpf). We observed that FGF2 significantly increased the number of ossified vertebrae in larval zebrafish at 7 days post-fertilization (dpf), while meclozine delayed vertebral ossification in FGF2-induced zebrafish. Meclozine also reversed the FGF2-induced upregulation of ossified craniofacial bone area, including ceratohyal, hyomandibular, and quadrate. The current study provided additional evidence regarding the inhibitory effect of meclozine on the FGF2-induced upregulation of MAPK signaling in chondrocytes and FGF2-induced development of craniofacial and vertebral bones.

## Introduction

Achondroplasia (ACH) is a common skeletal dysplasia with short-limbed short stature caused by gain-of-function mutations in fibroblast growth factor receptor 3 (*FGFR3*) (Rousseau et al., 1994; Shiang et al., 1994). In addition to short stature, ACH is associated with frontal bossing, midface hypoplasia, lumbar lordosis, limited elbow extension, and trident hand (Horton et al., 2007). Furthermore, foramen magnum stenosis can sometimes lead to serious complications, such as hydrocephalus, central sleep apnea, and sudden death (Hecht et al., 1989). Most adult patients with ACH exhibit spinal canal stenosis (Fredwall et al., 2020). Stenoses of the foramen magnum and spinal canal can develop owing to the premature closure of the synchondroses of the cranial base and vertebral arch (Hecht et al., 1989; Horton et al., 2007). In contrast, loss-of-function mutations in *FGFR3* cause camptodactyly, tall stature, scoliosis, and hearing loss syndrome (Toydemir et al., 2006). Thus, FGFR3 is a negative regulator of endochondral bone growth.

Previously, we demonstrated that meclozine, which has been employed as an anti-motion sickness treatment for more than 50 years, ameliorated the FGF2-mediated suppression of proliferating rat chondrosarcoma (RCS) cells by attenuating FGFR3 signaling (Matsushita et al., 2013). Oral administration of meclozine also increased bone elongation in a mouse model of ACH (*Fgfr3* ^ach^ mice) (Matsushita et al., 2015; Matsushita et al., 2017a). However, foramen magnum stenosis was not improved following meclozine administration from postnatal day 7. Conversely, maternal administration of meclozine partially rescued premature synchondrosis closure in *Fgfr3* ^ach^ mouse embryos (Matsushita et al., 2017b), but the effect of meclozine on craniofacial bone development was not significant, probably due to the low rate of placental drug transport. The effect of early meclozine administration on craniofacial bone development in ACH remains unclear. During craniofacial skeletogenesis, bone morphogenic protein (BMP) signaling is an early inductive signal required for cell migration, condensation, proliferation, and differentiation (Nie et al., 2006). In addition to accelerated endochondral ossification, FGFR3 signaling in chondrocytes increases the expression of BMP ligands and decreases the expression of BMP antagonists via the mitogen-activated protein kinase (MAPK) pathway (Matsushita et al., 2009). Reportedly, decreased secretion of BMP antagonists results in enhanced osteoblastogenesis in a paracrine manner. Accordingly, inhibition of the MAPK pathway in chondrocytes could attenuate craniofacial bone development.

Zebrafish have been used to examine the pathobiology of various diseases, including cardiovascular diseases (Priya et al., 2020), neurological disorders (Escamilla et al., 2017), and cancer (Hosono et al., 2017). Zebrafish have been employed to investigate bone and cartilage development, as phenotypes can be analyzed from the embryonic stage by exploiting the short development time (Hammond and Moro, 2012). As observed in humans, bone growth in zebrafish is composed of both intramembranous and endochondral ossifications (Weigele and Franz-Odendaal, 2016). Notably, *Fgfr3* was expressed in both osteoblasts and chondrocytes in zebrafish, similar to that observed in higher vertebrates (Ledwon et al., 2018). Therefore, zebrafish can be used to evaluate the effect of FGFR3 signaling on bone development between the embryonic and postnatal stages.

The purpose of the current study was to evaluate the effect of meclozine on vertebral and craniofacial bone development. We hypothesized that meclozine could inhibit FGF2-induced activation of MAPK pathway followed by downstream signalings including BMPs in chondrocyte, and attenuate FGF2-treated vertebral and craniofacial bone development. In the current study, we evaluated the inhibitory effect of meclozine on the MAPK pathway in embryonic murine cartilage. In addition, we analyzed the effect of FGF2 administration on bone and cartilage in larval zebrafish, as well as the effect of meclozine administration on early bone development in FGF2-treated larval zebrafish.

## Results

### Meclozine Attenuates MAPK Signaling in Mice FGF2-Treated Embryonic Tibia

**FIGURE 1:**
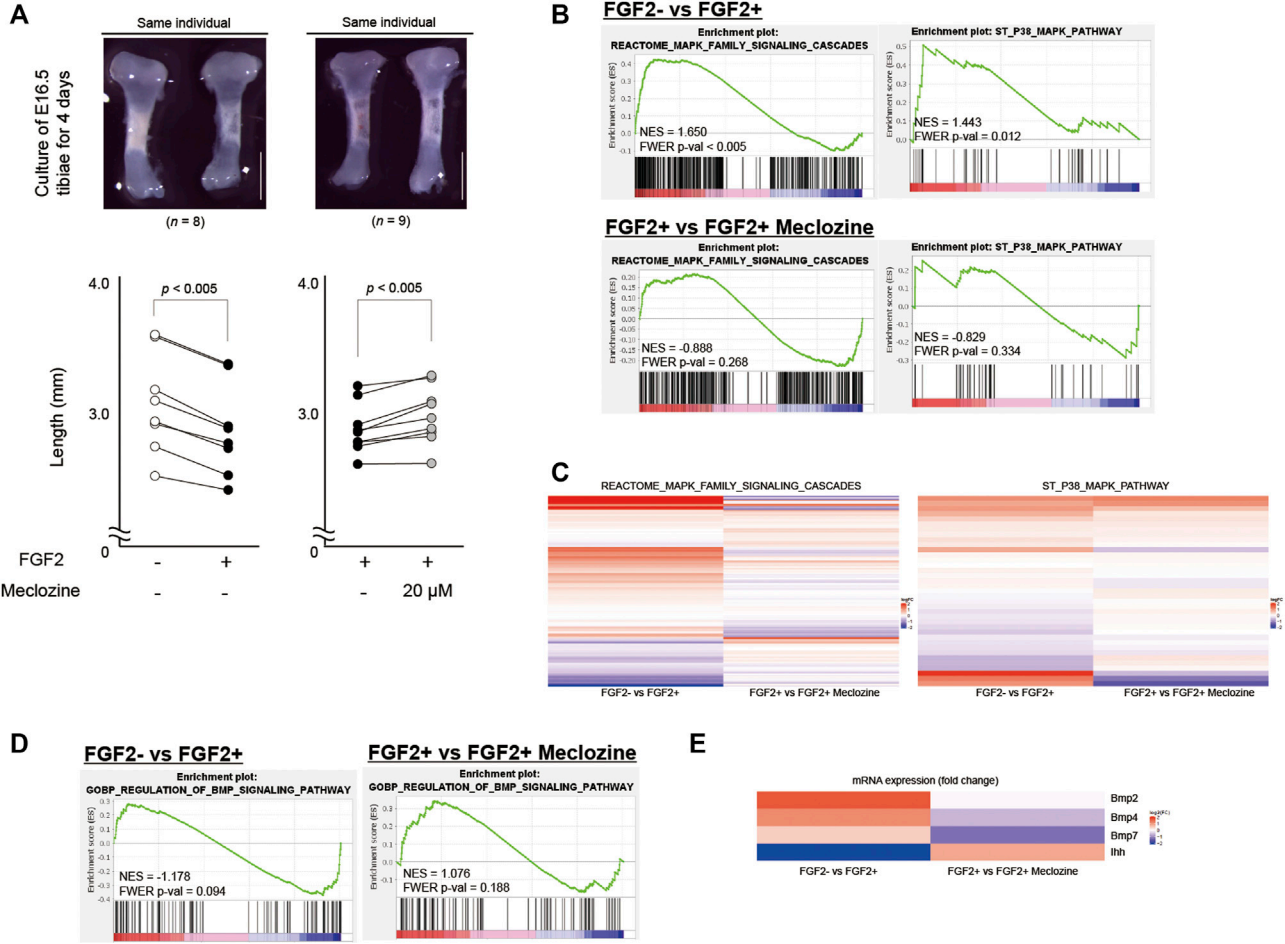
Meclozine attenuates the MAPK pathway of FGF2-treated tibiae in the organ culture system. **(A)** Upper panels: Representative images of E16.5 tibiae of wild-type mice after 4-day culture. Scale bares indicate 1 mm. Lower panels: Absolute bone length after 4-day treatment. Dots indicate the length. Lines are drawn between dots of the same individual. Statistical significance was analyzed by paired Student’s *t*-test. **(B)** Enrichment plots of two MAPK signaling-associated gene sets identified by GSEA between FGF2- and FGF2+, and FGF2+ and FGF2+ meclozine in the articular cartilage of *ex vivo* cultured tibiae. **(C)** Heatmap depicting the expression of the genes in REACTOME_MAPK_FAMILY_SIGNALING_CASCADES signature and ST_P38_MAPK_PATHWAY signature between FGF2- and FGF2+, and FGF2+ and FGF2+ meclozine in the articular cartilage of *ex vivo* cultured tibiae. **(D)** Enrichment plots of BMP signaling-associated gene set identified by GSEA between FGF2- and FGF2+, and FGF2+ and FGF2+ meclozine in the articular cartilage of *ex vivo* cultured tibiae. **(E)** Heatmap depicting the expression of the *Ihh*, *Bmp2*, *Bmp4*, and *Bmp7* between FGF2- and FGF2+, and FGF2+ and FGF2+ meclozine in the articular cartilage of *ex vivo* cultured tibiae. MAPK, mitogen-activated protein kinase; GSEA, Gene set enrichment analysis.

As previously reported (Matsushita et al., 2013), we cultured FGF2-treated embryonic tibiae of wild-type mice with or without meclozine for 4 days. Untreated tibiae were found to be elongated compared to FGF2-treated tibiae by 7.3% (*p* < 0.005). However, meclozine enhanced the bone length of FGF2-treated tibiae by 3.8% (*p* < 0.005) (Figure 1A). To more broadly assess the signaling pathways affected by meclozine treatment, differential expression was analyzed by RNA-seq using FGF2-treated tibiae, with or without meclozine in the cartilage harvested from cultured tibiae. Gene set enrichment analysis (GSEA) (Subramanian et al., 2005) revealed significant enrichment of the MAPK pathway (REACTOME_MAPK_FAMILY_SIGNALING_CASCADES) and its subset p38 pathway (ST_P38_MAPK_PATHWAY), as well as non-significant enrichment of the MEK-ERK pathway (REACTOME_MAPK3_ERK1_ACTIVATION), following FGF2 treatment (Figure 1B and Supplementary Figure S1). In contrast, meclozine co-treatment partially suppressed a subset of genes upregulated by FGF2 treatment (Figures 1B,C, and Supplementary Figure S1). Furthermore, neither FGF2 nor meclozine affected the enrichment score of the c-Jun N-terminal kinase (JNK) pathway (GCM_MAPK10) (Supplementary Figure S1).

FGF2 or meclozine treatment did not result in significant change in BMP pathway (GOBP_REGULATION_OF_BMP_SIGNALING_PATHWAY) (Figure 1D). FGF2, however, downregulated *Indian hedgehog* (*Ihh*) and upregulated *Bmp2*, *Bmp4*, and *Bmp7* while meclozine reversed these expression levels (Figure 1E). We recapitulated these results by qRT-PCR (Supplementary Figure S2).

### Optimal Doses of FGF2 and Meclozine Are Determined for Larval Zebrafish

**FIGURE 2:**
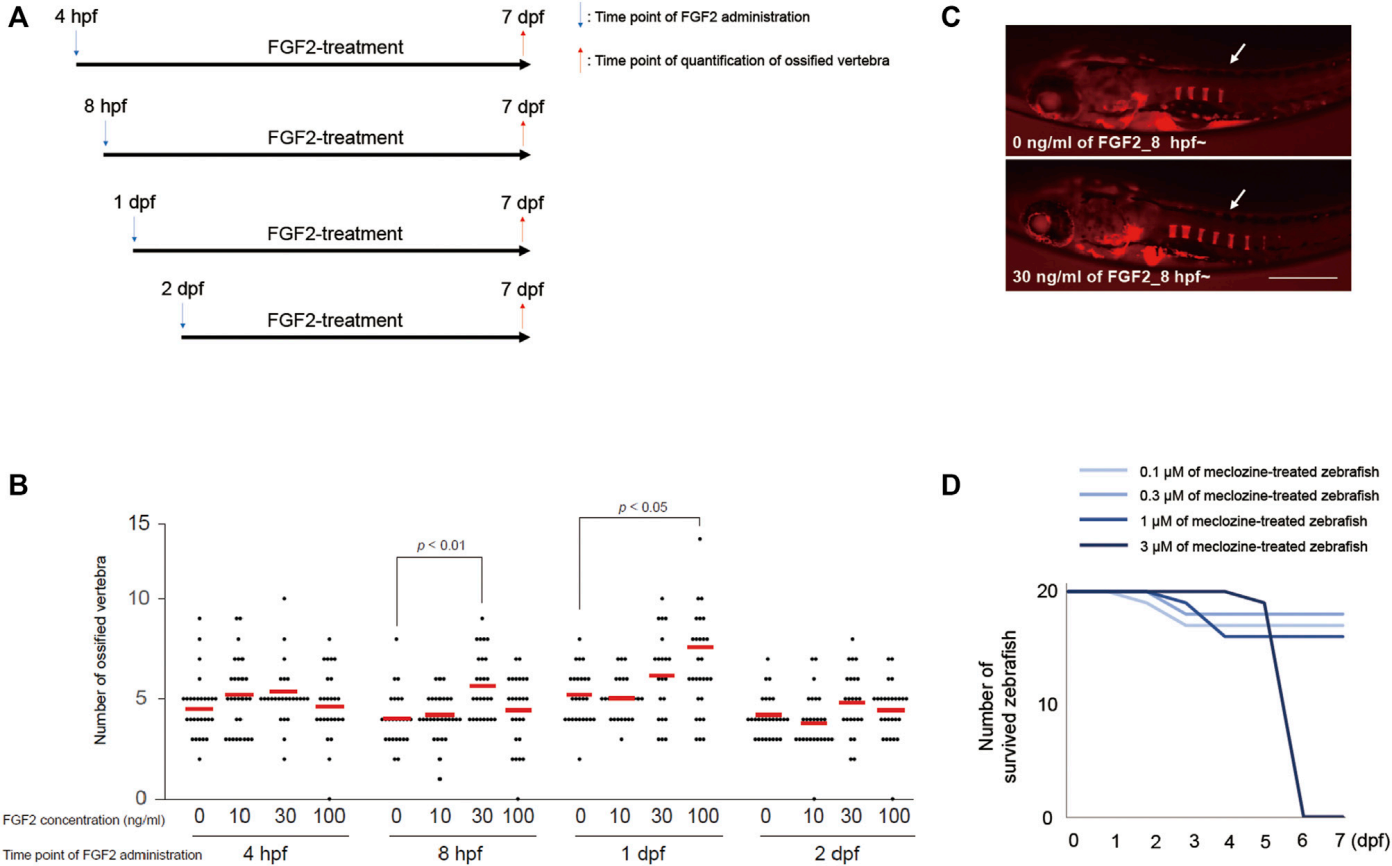
Treatment protocol of FGF2 and meclozine are determined for evaluating vertebral ossification in larval zebrafish. **(A)** Treatment regimen of FGF2 for larval zebrafish. **(B)** Quantification of ossified vertebrae after FGF2 treatment. Dots indicate the number of ossified vertebrae of each sample, and bars indicate means. Statistical significance was analyzed by one-way ANOVA with post-hoc Tukey HSD. **(C)** Representative images of larval zebrafish from the lateral view at seven dpf stained with Alizarin red after 30 ng/mL FGF2 treatment from eight hpf to seven dpf. Arrows: ossified vertebrae. Scale bar indicates 500 µm. **(D)** Survival curve of larval zebrafish treated with each dose of meclozine from eight hpf to seven dpf.

We initially examined the temporal profiles of the effect of FGF2 on vertebral ossification in larval zebrafish. The embryos were treated with each dose of FGF2 from 4 h post-fertilization (hpf), eight hpf, 1 day post-fertilization (dpf), or two dpf; the ossified bones were visualized by staining with Alizarin red at seven dpf (Figure 2A). We quantified the number of ossified vertebrae from the lateral view of the larval zebrafish. Further, we selected 30 ng/mL FGF2 treatment from eight hpf owing to a significant increase in the number of ossified vertebrae in larval zebrafish at seven dpf (Figures 2B,C). To determine the maximum tolerable meclozine dose, we added 0.1, 0.3, 1, or 3 μM of meclozine to larval zebrafish from eight hpf to seven dpf and observed no surviving larval zebrafish following treatment with 3 μM meclozine at the established endpoint (Figure 2D). Thus, we chose 1 μM meclozine as the maximum tolerated dose.

### Meclozine Delays FGF2-Induced Spinal and Craniofacial Ossification in Larval Zebrafish

**FIGURE 3:**
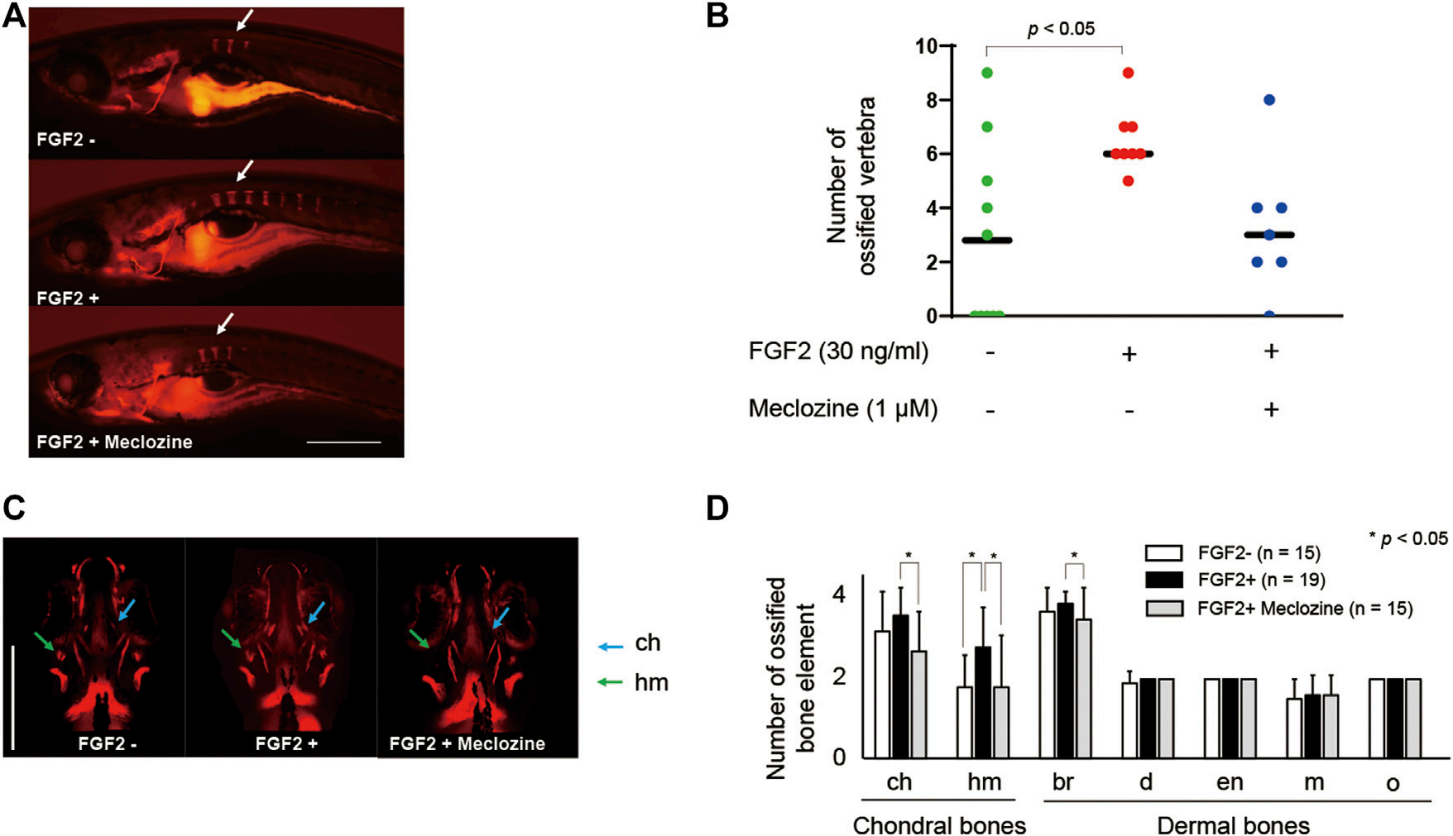
Meclozine attenuates spinal and craniofacial bone ossification in FGF2-treated larval zebrafish. **(A)** Representative images of larval zebrafish from the lateral view at seven dpf stained with Alizarin red after 30 ng/mL FGF2 treatment, with or without 1 µM meclozine, from eight hpf to seven dpf. Arrows: ossified vertebrae. Scale bar indicates 500 µm. **(B)** Quantification of ossified vertebrae after FGF2 treatment, with or without meclozine. Dots indicate the number of ossified vertebrae of each sample, and bars indicate means. Statistical significance was analyzed by one-way ANOVA with post-hoc Tukey HSD. hpf, hours post-fertilization; dpf, days post-fertilization. **(C)** Representative craniofacial bone elements of larval zebrafish from anteroposterior view at seven dpf stained with Alizarin red after FGF2 treatment, with or without meclozine. Scale bar indicates 500 µm. **(D)** Quantification of the number of each ossified craniofacial bone element, including ceratohyal (ch), hyomandibular (hm), branchiostegal ray (br), dentary (d), entopterygoid (en), maxilla (m), and opercle (o), after FGF2 treatment with or without meclozine. Data values are presented as means and standard deviation (SD). Statistical significance was analyzed by one-way ANOVA with post-hoc Tukey HSD.

Treatment with 1 μM meclozine attenuated vertebral ossification in larval zebrafish enhanced by FGF2 treatment (Figure 3A). Quantitative analyses revealed that FGF2 significantly increased the number of ossified vertebrae in larval zebrafish, whereas meclozine almost normalized the number of ossified vertebrae in FGF2-treated larval zebrafish (Figure 3B). We also evaluated the effect of meclozine on FGF2-induced craniofacial bone hyperossification by quantifying the number of ossified bone elements, including ceratohyal (ch), hyomandibular (hm), branchiostegal rays (br), dentary (d), entopterygoid (en), maxilla (m), and opercle (o) (Supplementary Figure S3A). FGF2 enhanced craniofacial ossification in larval zebrafish, whereas meclozine suppressed FGF2-induced ossification (Figure 3C). The number of ossified ch, hm, and br was significantly reduced following meclozine treatment in FGF2-induced larval zebrafish (Figure 3D). We confirmed the replicability of the effect of meclozine on FGF2-treated spinal and craniofacial bones in an additional experiment (Supplementary Figures S4A–C).

### Meclozine Reverses FGF2-Induced Acceleration of Endochondral Ossification in Larval Zebrafish

**FIGURE 4:**
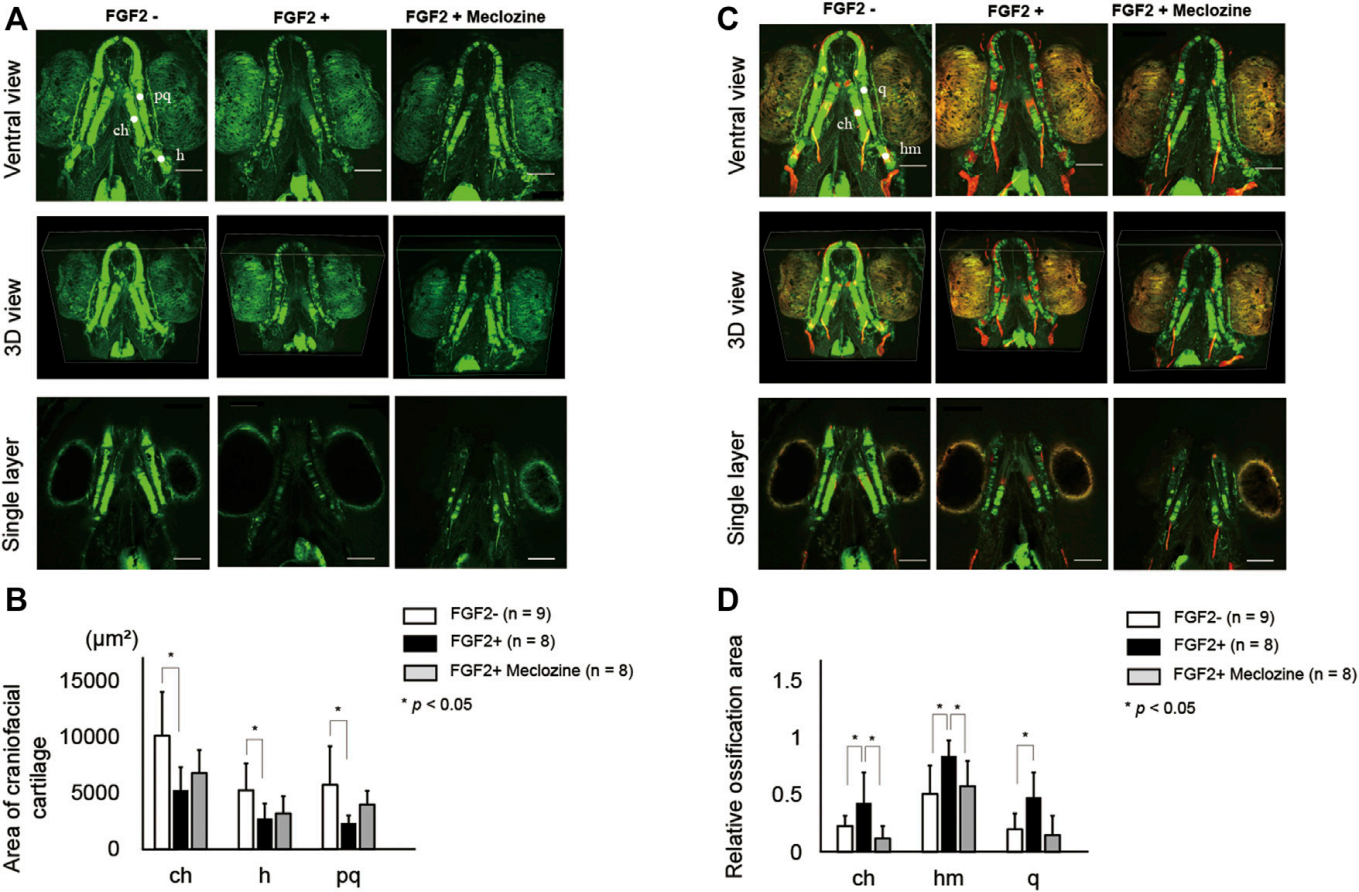
Meclozine ameliorates FGF2-induced hyper ossification in larval zebrafish. **(A)** Representative craniofacial cartilage elements of larval zebrafish from ventral view, three-dimensional (3D) view, and single layer at seven dpf in Tg (col2a1a:EGFP) after FGF2 treatment, with or without meclozine. Scale bar indicates 100 µm. **(B)** Quantification of area of craniofacial cartilage, including ceratohyal (ch), hyosymplectic (h), and palatoquadrate (pq) after FGF2 treatment with or without meclozine. **(C)** Representative craniofacial cartilage and bone elements of larval zebrafish from ventral view, 3D view, and single layer at seven dpf in Tg (col2a1a:EGFP) stained with Alizarine red after FGF2 treatment, with or without meclozine. Scale bar indicates 100 µm. **(D)** Quantification of relative ossification area, including ceratohyal (ch), hyomandibular (hm), and quadrate (q) after FGF2 treatment with or without meclozine. Relative ossification area was calculated by dividing each red signal area by each green area. Data values are presented as means and standard deviation (SD). Statistical significance was analyzed by one-way ANOVA with post-hoc Tukey HSD.

Both FGF2 alone and FGF2 + meclozine failed to exert a significant effect on chondrocyte shape in conventional wide-field images (Supplementary Figure S5A). FGF2 and meclozine did not alter the measured lengths of the anterior limit (an)-ethmoid plate (et), an-posterior limit (po), et-po, articulation (ar)-ar, ceratohyal (ch)-ch, and hyosymplectic (h)-h, as well as the jaw angle (Supplementary Figures S3B,C, and Supplementary Figures S4B,C). However, confocal images indicated that FGF2 downregulated the signals of craniofacial cartilage (Figure 4A). Meclozine ameliorated FGF2-induced atrophy of the cartilage. Areas of craniofacial cartilage in ch, h, and palatoquadrate (pq) were significantly decreased by FGF2 treatment, while there were no statistical differences of these areas between FGF2- and FGF2 + meclozine (Figure 4B). On the other hand, craniofacial bones were hyperdeveloped by FGF2 treatment and meclozine counteracted the effect of FGF2 in confocal images (Figure 4C). Quantitative analyses indicated that meclozine reduced the FGF2-induced ossification of craniofacial bones, including ch, hm, and quadrate (q) (Figure 4D).

## Discussion

Herein, we revealed that meclozine counteracted growth retardation in FGF2-induced embryonic tibiae by attenuating the MAPK family signaling cascades activated by FGF2 treatment in chondrocytes. The effect of FGF2 on accelerating vertebral and craniofacial bone development in larval zebrafish was reversed following meclozine administration. Such results indicate that meclozine neutralizes the effect of FGF2 on abnormal bone and cartilage development.

A recent study reported that *Fgfr3*-deficient zebrafish showed craniofacial malformations associated with bone mineralization defects, abnormal hypertrophy, and disarrangement in the growth plate, similar to human CATSHL syndrome (Sun et al., 2020). In another study, zebrafish carrying loss-of-function of *Fgfr3* demonstrated craniofacial abnormalities, including microcephaly, Wormian bone formation, and small frontal and parietal bones with delayed osteoblast genesis (Dambroise et al., 2020). These results indicate that FGFR3 is a positive regulator of intramembranous ossification. According to the findings of the current study, FGF2, an FGFR3 ligand, had a positive impact on craniofacial development, which opposed the defective bone development observed in *Fgfr3*-deficient zebrafish. Reportedly, NVP-BGJ398, an FGFR tyrosine kinase inhibitor, enhances bone growth in a mouse model with a gain-of-function mutation in *Fgfr3* (Komla-Ebri et al., 2016). Dambroise et al. (Dambroise et al., 2020) demonstrated that BGJ398 inhibited cranial vault development in zebrafish. Similar results would be obtained after long-term treatment of meclozine in zebrafish. In addition to enhancing longitudinal bone growth, FGFR3 inhibitors can lead to delayed bone ossification during early embryo life.

RCS cells have been utilized as an *in vitro* model of ACH, as FGFR3 is abundantly expressed in RCS cells, and FGF2 inhibits cell proliferation and differentiation (Krejci et al., 2010). In the embryonic bone culture system, longitudinal bone growth is attenuated following FGF2 treatment (Krejci et al., 2010; Gudernova et al., 2016). These alterations in both RCS cells and cultured bone cells *via* continuous stimulation of FGF2 were reversed following meclozine administration (Matsushita et al., 2013). The current study also provides evidence of the inhibitory effect of meclozine on FGF2-induced bone hyperossification in larval zebrafish.

Activated FGFR3 signaling upregulated MAPK, including extracellular signal-regulated kinase (ERK), p38, and JNK, while FGFR3 in growth plate chondrocytes activated ERK and p38 (Ornitz and Marie, 2019). GSEA indicated that meclozine attenuated the MAPK family signaling cascades. Previously, we reported that meclozine inhibits ERK phosphorylation in FGF2-treated RCS cells (Matsushita et al., 2013). Additionally, Guo et al. (Guo et al., 2017) have indicated that meclozine attenuates ERK phosphorylation augmented by the receptor activator of nuclear factor-κB ligand (RANKL) in bone marrow-derived macrophages (BMMs). They further demonstrated that meclozine inhibited p38 phosphorylation in RANKL-treated BMMs. Thus, the simultaneous inhibition of ERK and p38 phosphorylation suggests that the molecular target of meclozine might be at the level of MAPKKK or higher (Chao et al., 1999; Fritz et al., 2006; Craig et al., 2008). However, JNK phosphorylation was not inhibited by meclozine in RANKL-treated BMMs (Guo et al., 2017). Similarly, FGF2 does not induce JNK phosphorylation in RCS cells (Krejci et al., 2004). GSEA also revealed that neither FGF2 nor meclozine had a positive effect on the JNK pathway.

St-Jacques et al. (St-Jacques et al., 1999) demonstrated that *Ihh* had an essential role of osteoblast development in endochondral bones. *Ihh* and *Bmp4* in a growth plate cartilage were suppressed in a mouse model with gain-of-function mutation of FGFR3 (Naski et al., 1998). Current results also indicated that *Ihh* did not seem to be mediated in FGF2-induced bone formation due to the downregulation of *Ihh* by FGF2 treatment. On the other hand, *Bmp2*, *Bmp4*, and *Bmp7* in chondrocytes were upregulated by FGF2 treatment. *Bmp2* and *Bmp7* were similarly promoted in FGF18-treated chondrocytes (Matsushita et al., 2009). However, expression of *Bmp2*, *Bmp4*, and *Bmp7* in chondrocytes were increased in a conditional knock-out mouse of FGFR3 (Wen et al., 2016). Although there seemed to be physiological difference between FGF2-treated chondrocytes and FGFR3-upregulated chondrocytes, meclozine definitely reversed FGF2-induced regulation of *Ihh* and *Bmp*s in chondrocytes.

The present study had several limitations. First, we employed FGF2-treated zebrafish instead of zebrafish with a gain-of-function mutation in *Fgfr3*. The phenotype could be similar between both zebrafish since the longitudinal bones were shortened in a FGF2-overexpressing mouse model (Coffin et al., 1995) as well as an ACH mouse model (Naski et al., 1998), and expression pattern of zebrafish *fgfr3* was also similar to the expression observed in higher vertebrates (Ledwon et al., 2018). We did not directly determine the effect of meclozine on craniofacial development in the ACH zebrafish model, although we demonstrated that meclozine neutralized the FGF2-induced phenotype. Second, we observed the phenotype of FGF2-treated zebrafish at seven dpf; this is because zebrafish cannot be grown in a culture plate after seven dpf. Thus, another phenotype could have existed in older zebrafish under FGF2 treatment. Lastly, RNA-seq was performed using cartilage harvested from murine tibiae. Nonetheless, the limitation of the current study lies in the lack of relevant animal experiments for further verification.

Collectively, our findings revealed that meclozine attenuates FGF2-induced craniofacial bone development in larval zebrafish. However, further studies are needed to evaluate the long-term effects of early meclozine administration.

## Materials and Methods

### Bone Explant Culture

All mouse studies were approved by the Animal Care and Use Committee of the Nagoya University. The tibiae of wild-type mice (C57BL/6 background) (Japan SLC) were dissected under a microscope on embryonic day 16.5. Thereafter, tibiae were cultured in a 48-well plate with BGJb medium (12591038, Invitrogen) supplemented with 0.2% bovine serum albumin and 150 mg/mL ascorbic acid. FGF2 (3339-FB-025, R and D Systems) was administered in the presence or absence of 20 µM meclozine (155341, MP Biomedicals) for 4 days. The medium was changed daily. The longitudinal length of the bone, defined as the length between the proximal and distal articular cartilage, was measured using ImageJ software (U. S. National Institutes of Health, Bethesda, MD, USA) (Matsushita et al., 2013; Matsushita et al., 2015).

### Zebrafish Maintenance

All zebrafish studies were approved by the Animal Care and Use Committee of the Aichi Cancer Center Research Institute. Zebrafish (*Danio rerio*) were maintained at 28–29°C under a 14 h light:10 h dark cycle in a filtered freshwater recirculation system and fed three times daily, as described previously (Westerfield, 2007). Breeding was performed approximately 90 dpf when fish were sexually mature. Previously separated male and female zebrafish were introduced into a breeding tank, and eggs were collected. Embryos were raised at 28.5°C and staged in hpf, according to standard procedures. The AB strain, originally obtained from Zebrafish International Resource Center (ZIRC), was used to generate the transgenic lines described in this study.

### Generation of Tg (col2a1a:EGFP) Zebrafish

The plasmid col2a1a:EGFP was constructed using the Tol2/Gateway kit (Kwan et al., 2007). The col2a1a promoter was amplified using gDNA extracted from embryos as a template and cloned into the p5ʹE entry vector. The EGFP and polyA tails were cloned into the pME and p3ʹE entry vectors, respectively. These were then assembled into the Tol2 destination vector using the MultiSite Gateway Technology system (Invitrogen). One-cell stage embryos were microinjected with 50 ng/μL Tol2 mRNA and 50 ng/μL of col2a1a:EGFP construct using a pneumatic pico-pump (PV-820, World Precision Instrument). F0 zebrafish were crossed with wild-type AB to generate F1 embryos, which were screened for GFP expression. F0 zebrafish that could produce germ-line GFP expression were crossed to produce F1 heterozygotes. F2 generations were generated by F1 heterozygotes in-cross.

### Preparation of Recombinant Protein

His-tagged zebrafish-fgf2 (zFGF2) protein was expressed in Sf9 insect cells using a Gateway system (Invitrogen), according to the manufacturer’s instructions, followed by purification using imidazole (19004-22, Nacalai, Japan)-affinity chromatography.

### Zebrafish Embryo Treatment

F2 heterozygous col2a1a:EGFP zebrafish were crossed with wild-type zebrafish to produce heterozygous embryos. Embryos were harvested at indicated stages and cultured in 6-well plates with zFGF2, with or without meclozine, until seven dpf. Larvae were fed Dried Rotifer Sheet (HIKARI-LABO Hirugata-wamushi, KYORIN CO., LTD., Japan [Nakayama et al., 2018)] at five dpf. *In vivo* skeletal staining was performed with 0.05% Alizarin red (A5533, Sigma-Aldrich) in E3 medium for 60 min and subsequently washed with E3 medium. Zebrafish larvae were anesthetized with 0.016% tricaine (T0941, Tokyo Chemical Industry Co., Ltd., Japan), and images were captured with a Keyence BZ-X810 All-in-One fluorescence microscope (Keyence, Osaka, Japan). The number of ossified vertebrae and bone elements was counted using skeletal images stained with Alizarin red. Vertebrae and craniofacial bone elements with any staining of Alizarin red was counted as ossified bones (Felber et al., 2011). From images visualized using EGFP, we identified each cartilaginous element, including mk, pq, h, an, et, po, ar, and ch (Supplementary Figure S2B). The jaw angle was defined as the angle between the bilateral channels (Supplementary Figure S2C). Larval zebrafish visualized by EGFP and stained with Alizarin red were also captured with TiE-A1R confocal microscope (Nicon, Tokyo, Japan) to reconstruct three-dimensional (3D) images. The length, angle, and area of interest were calculated using the ImageJ software. The number of zebrafish used in the current study was indicated in Supplementary Table S1.

### RNA Isolation

Total RNA was isolated from the cartilage of the cultured tibiae using miRNeasy Mini Kit (217004, QIAGEN) with DNase I (79254, QIAGEN) digestion according to the manufacturer’s instructions. RNA integrity was verified using an Agilent Bioanalyzer 2100 (Agilent Technologies, Palo Alto, CA, USA).

### Quantitative Real-Time

qRT-PCR was performed using Power SYBR Green Mastermix (Applied Biosystems, CA) on an Applied Biosystems 7900HT Real-Time PCR System. All oligonucleotide primers were obtained from FASMAC (Kanagawa, Japan) and are listed in Supplementary Table S2. The housekeeping gene, Gapdh, was amplified as controls. Fold changes were calculated relative to Gapdh and normalized to the median value of the FGF2+ samples.

### PCR RNA-Sequencing

RNA-seq was performed by Filgen Inc. (Nagoya, Japan) in paired-end mode. RNA-sequencing reads were aligned to the mouse reference genome (mm9) using HISAT2 (Kim et al., 2015) and converted to gene counts using featureCounts (Liao et al., 2014). The raw read counts were normalized using DESeq2 (Love et al., 2014) to estimate gene expression.

### Differential Expression Analysis and Gene Set Enrichment Analysis

GSEA was performed using the MSigDB v7.2. A family-wise error rate (FWER) *p*-value < 0.01 was considered significant.

### Statistical Analysis

All data are expressed as the mean and standard deviation. Statistical differences were determined using one-way analysis of variance (ANOVA) with post-hoc Tukey HSD and Student’s t-test. Data analysis was performed using IBM SPSS Statistics version 27 (IBM, Armonk, NY, United States). Statistical significance was set at *p* < 0.05.

## Data Availability

The datasets presented in this study can be found in online repositories. The names of the repository/repositories and accession number(s) can be found below: DNA Data Bank of Japan (DDBJ) (the accession number: PRJDB11737).
