# Supplementary material for: Meclozine Attenuates the MARK Pathway in Mammalian Chondrocytes and Ameliorates FGF2-Induced Bone Hyperossification in Larval Zebrafish
Source: Front Cell Dev Biol. 2022 Jan 18;9:694018. doi: 10.3389/fcell.2021.694018 (PMC8804316; doi:10.3389/fcell.2021.694018)
Supplement: Supplementary file 1 [file Table1.DOCX]

| **Supplementary Table 1 \| Number of zebrafish used in the different studies** | |
| --- | --- |
| **Study** | **Number of zebrafish** |
| Determining time point of FGF2 administration (4 hpd, 8 hpd, 1 dpf, and 2 dpf) and FGF2 concentration (0, 10, 30, and 100 ng/ml) | 411 |
| Determining meclozine concentration (0.1, 0.3, 1, and 1 µM) | 80 |
| First round of analysis for spinal bone ossification (FGF2-, FGF2+, and FGF2+ meclozine) | 25 |
| First round of analysis for craniofacial bone ossification (FGF2-, FGF2+, and FGF2+ meclozine) | 49 |
| Second round of analysis for spinal bone ossification (FGF2-, FGF2+, and FGF2+ meclozine) | 41 |
| Second round of analysis for craniofacial bone ossification (FGF2-, FGF2+, and FGF2+ meclozine) | 51 |
| Measuring the lengths among craniofacial cartilaginous elements (FGF2-, FGF2+, and FGF2+ meclozine) | 29 |
| Measuring jaw angle (FGF2-, FGF2+, and FGF2+ meclozine) | 44 |
| Analyses for craniofacial cartilage and bone using confocal images (FGF2-, FGF2+, and FGF2+ meclozine) | 25 |
