# Supplementary figures and images for: Meclozine Attenuates the MARK Pathway in Mammalian Chondrocytes and Ameliorates FGF2-Induced Bone Hyperossification in Larval Zebrafish
Source: Front Cell Dev Biol. 2022 Jan 18;9:694018. doi: 10.3389/fcell.2021.694018 (PMC8804316; doi:10.3389/fcell.2021.694018)

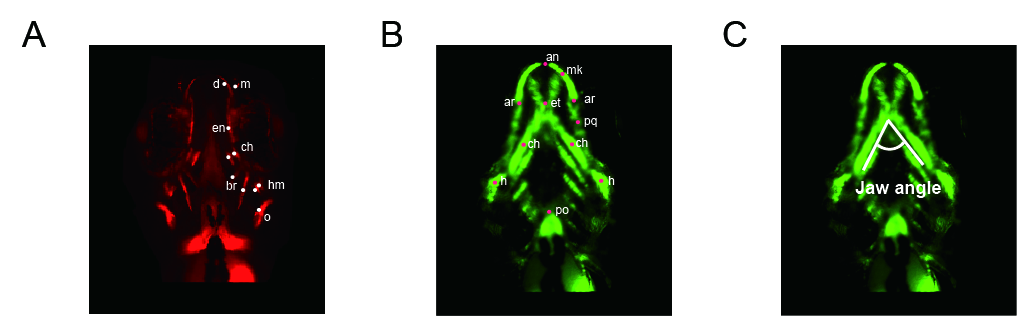

Supplement: Supplementary file 2 [file Image3.TIF]

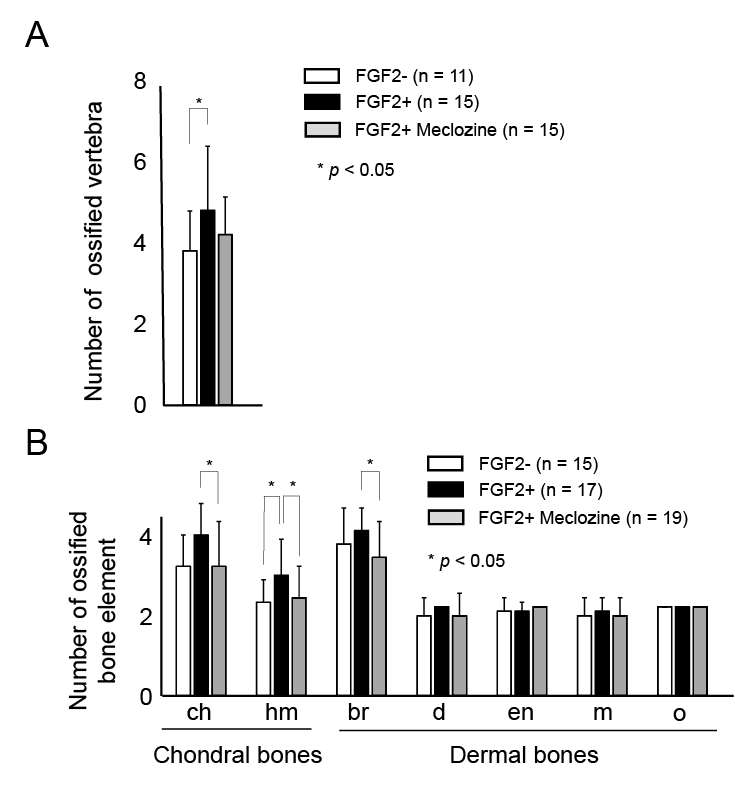

Supplement: Supplementary file 3 [file Image4.TIF]

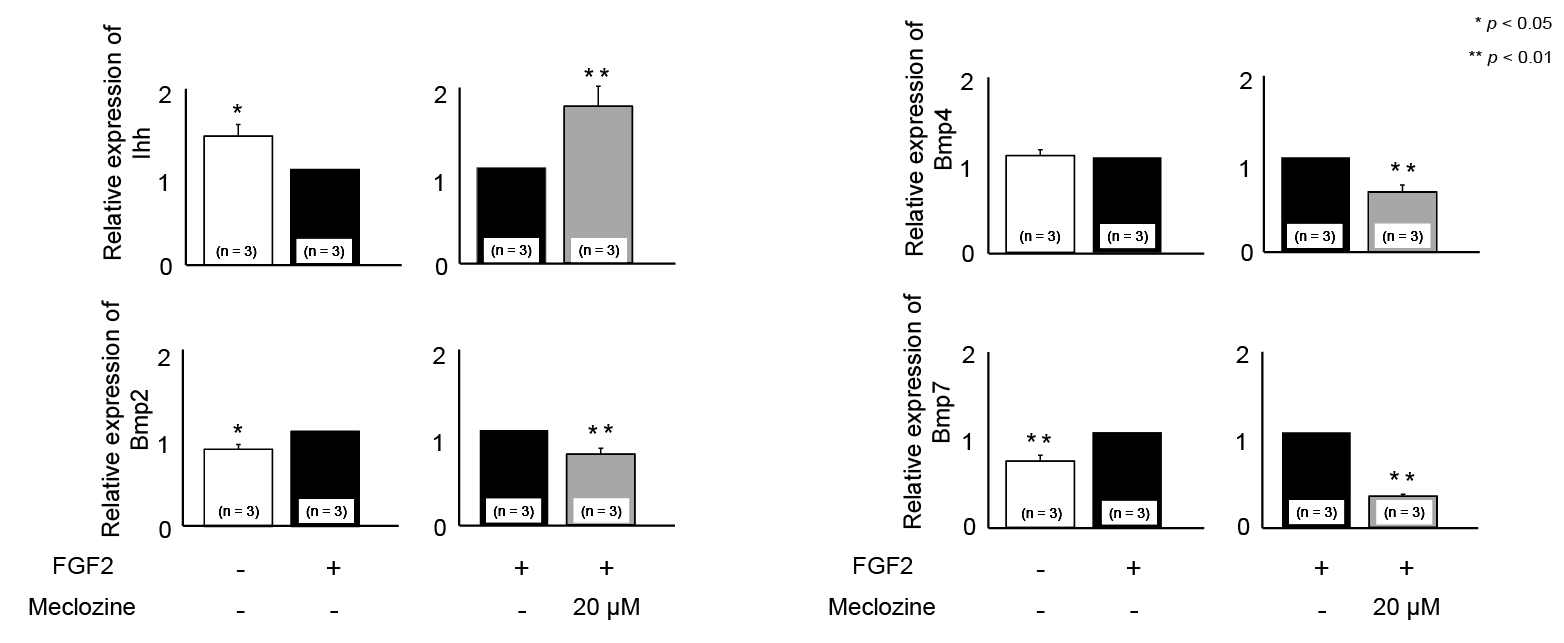

Supplement: Supplementary file 4 [file Image2.TIF]

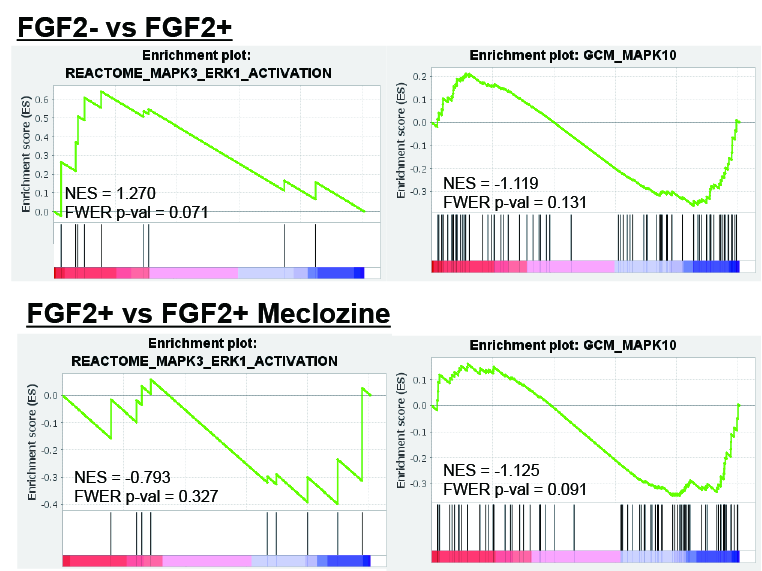

Supplement: Supplementary file 5 [file Image1.TIF]

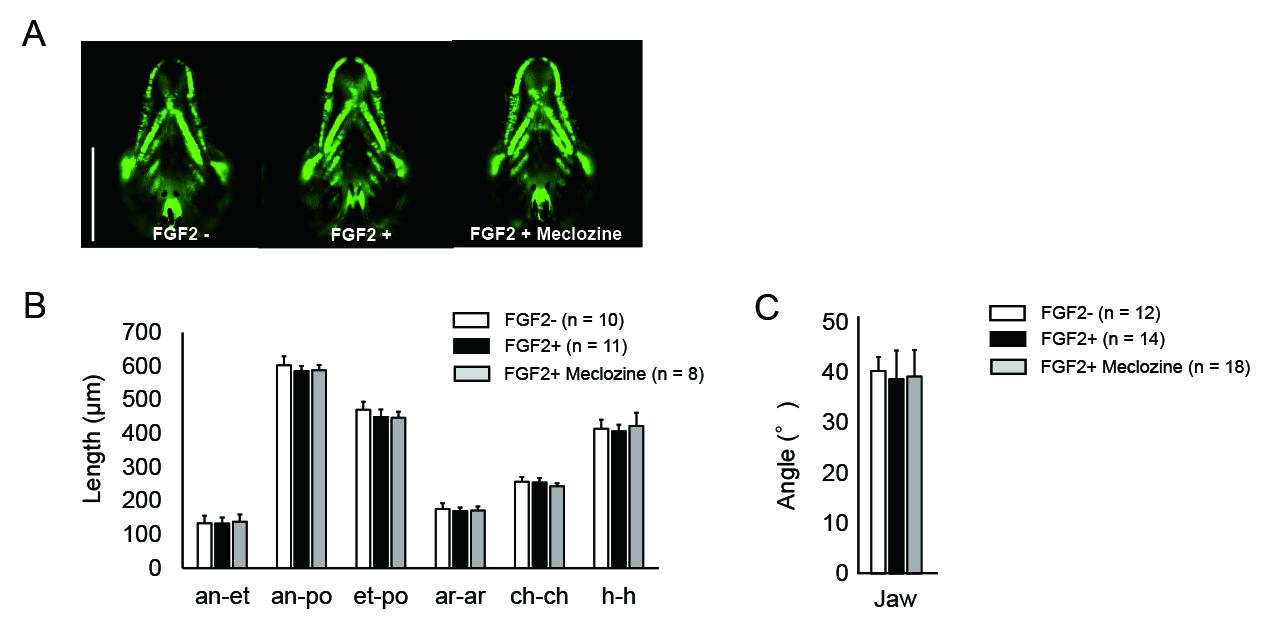

Supplement: Supplementary file 7 [file Image5.TIF]
