# Supplementary material for: Meclozine Attenuates the MARK Pathway in Mammalian Chondrocytes and Ameliorates FGF2-Induced Bone Hyperossification in Larval Zebrafish
Source: Front Cell Dev Biol. 2022 Jan 18;9:694018. doi: 10.3389/fcell.2021.694018 (PMC8804316; doi:10.3389/fcell.2021.694018)
Supplement: Supplementary file 6 [file Table2.DOCX]

| **Supplementary Table 2 \| Primers** | | |
| --- | --- | --- |
|  | **Forward (5'-3')** | **Reverse (5'-3')** |
| Gapdh | CCAATGTGTCCGTCGTGGATCT | GTTGAAGTCGCAGGAGACAACC |
| Ihh | TGGGACACTTGTGGTGGAGGA | AGGCGGTAGAGCATCTGAGGG |
| Bmp2 | TGAGGTGCACAGACTTGGA | TGTACGCGTGGAATGACCTA |
| Bmp4 | CTTCAACCTCAGCAGCATCC | GATGAGGTGTCCAGGAACCA |
| Bmp7 | GAGGGATCTTTACCGGCTCC | GTTGAAGAGGAAACGAAAAGCAG |
